# Genetics, Epigenetics, and Gender Impact in Axial-Spondyloarthritis Susceptibility: An Update on Genetic Polymorphisms and Their Sex Related Associations

**DOI:** 10.3389/fgene.2021.671976

**Published:** 2021-08-10

**Authors:** Maria Sole Chimenti, Carlo Perricone, Arianna D’Antonio, Mario Ferraioli, Paola Conigliaro, Paola Triggianese, Cinzia Ciccacci, Paola Borgiani, Roberto Perricone

**Affiliations:** ^1^Rheumatology, Allergology and Clinical Immunology, Department of Systems Medicine, University of Rome Tor Vergata, Rome, Italy; ^2^Rheumatology Unit, Department of Medicine and Surgery, University of Perugia, Perugia, Italy; ^3^Unicamillus, Saint Camillus International University of Health Sciences, Rome, Italy; ^4^Department of Biomedicine and Prevention, Genetics Unit, University of Rome “Tor Vergata”, Rome, Italy

**Keywords:** Spondyloarthritis, genetic predisposition and association, precision medicine, gender predisposition, gender medicine

## Abstract

Spondyloarthritis (SpA) is a group of chronic inflammatory rheumatic disease that can be divided into predominantly axial or predominantly peripheral involvement, with or without associated psoriasis, inflammatory bowel disease or previous infection. Axial SpA (axSpA) encompasses ankylosing spondylitis (AS) with radiological sacroiliitis, and a type without radiographic sacroiliitis, called “non-radiographic axial SpA” (nr-axSpA). Males and females show large differences in their susceptibility to SpA, such as distinctions in clinical patterns, phenotypes and in therapeutical response, particularly to TNF inhibitors (TNFi). Several studies indicate that AS women have doubled risk to failure TNFi compared with males. This diversity in drugs’ efficacy among women and men may be caused by differences in the balance of sex hormones and in gene-specific expression likely triggered by X-chromosome instability and gene-specific epigenetic modifications. Evidence reported that polymorphisms in microRNAs on X- and other chromosomes, such as miR-146a, miR-155, miR-125a-5p, miR-151a-3p and miR-22-3p, miR-199a-5p could be involved in the different clinical presentation of SpA, as well as disease activity. In addition, association with non−response to TNFi treatment and presence of IRAK3 and CHUCK genes in SpA patients was recently detected. Finally, polymorphisms in genes involved in IL-23/IL-17 pathway, such as in drug pharmacodynamics and pharmacokinetics may have a role in response to TNFi, IL17i, and IL23i. A major understanding of genomic variability could help in the development of new therapeutic targets or in taking advantages of different mechanisms of action of biological drugs. Moving from the multifactorial etiology of disease, the present review aims at evaluating genetic and epigenetic factors and their relationship with sex and bDMARDs response, helping to investigate the different expression among males and females of genes on X- and other chromosomes, as well as mi-RNA, to highlight relationships between sex and occurrence of specific phenotypes and symptoms of the disease. Moreover, the role of the epigenetic modification in relation to immune-regulatory mechanisms will be evaluated.

## Introduction

Spondyloarthritis (SpA) is a heterogeneous group of inflammatory chronic diseases characterized by common clinical features, as inflammatory back pain, peripheral joint involvement, dactylitis and enthesitis (Chimenti et al., 2020b). SpA can be divided into predominantly axial or predominantly peripheral form, with or without psoriasis (PsO), inflammatory bowel disease (IBD) or previous associated infection. Axial SpA (axSpA) encompasses ankylosing spondylitis (AS) with radiological signs of sacroiliitis, and a type without radiographic sacroiliitis, called “non-radiographic axial SpA” (nr-axSpA) (Chimenti et al., 2019a; Ward et al., 2019). All the clinical subtypes share common pathogenic, clinical, and radiologic features such as the genetic predisposition and the association with HLA-B27, the presence of extra-articular involvement and bone remodeling associated with bone resorption and osteoproductive lesions called syndesmophytes (Chimenti et al., 2020b). In axial-SpA the sex prevalence has dramatically changed during the last two decades: SpA were linked to male gender with a ratio of 10:1 with respect to female gender, nowadays the ratio has reduced to 3:1 for AS/axSpA (West, 1949). However, in contrast to AS, nr-axSpA male and female patients present the same prevalence (van der Horst-Bruinsma et al., 2013). Recent evidence supports the hypothesis of different clinical subtypes among males and females, suggesting a genetic and hormonal based pathogenesis. In this direction, the pathogenesis of SpA is not clearly understood to date and the role of immunological and genetic data showed clear sex dimorphisms in SpA patients (Rusman et al., 2018). Certainly, pathogenic mechanisms of SpA comprise a complex interplay among genetic background, environmental triggers, and mechanical stress that leads to the overall activation of inflammation and autoimmunity (Pedersen and Maksymowych, 2018). The role of genetic susceptibility in SpA was strongly associated with the presence of human leukocyte antigen (HLA) and non-HLA alleles that surely takes part in the predisposition to the disease (Brown et al., 2000). The class I HLA allele HLA–B27 is strongly linked with the development of SpA due to its role in the pathogenesis of the disease (Hacquard-Bouder et al., 2007). The revolution of the genome-wide association study (GWAS) era has identified hundreds of genes associated with SpA, mainly IL23R, ERAP1 (Endoplasmic Reticulum Aminopeptidase-1), ERAP2, and MEFV (Mediterranean fever) linked to innate and acquired immune response and cytokines production (Brown et al., 2000). The current interest in Precision Medicine, in order to identify preventive and therapeutic interventions, is increasing in the management of SpA patients. Clinical effect of biological DMARDs (bDMARDs) is now known to be affected by gender, as well as the clinical, genetic, and psychosocial life-style context (Garrido-Cumbrera et al., 2021). This different efficacy in women and men is due to biological differences which may be caused by sex-specific gene expression likely triggered by gene-specific epigenetic modifications. However, in SpA patients, a series of factors may interfere in epigenetic modifications: age, smoke, diet, and environmental factors (Chimenti et al., 2020b). In this context, the clinical phenotype and the response to drugs can be influenced by gender and considered as complex traits in SpA. The study of genetic and epigenetic mechanisms involved in the pathogenesis of SpA may be of help to define novel targets for more effective therapy. The aim of this review was to highlight the role of genetics and epigenetics in the susceptibility and clinical pattern of SpA as well as in bDMARDs treatment response variability with a focus on gender differences and predisposition.

## Genetic Predisposition to Spondyloarthritis

Over the past decades a growing interest in the pathogenesis of SpA has provided rapid advances in understanding the genetic basis of the disease. A family aggregation to SpA has long been recognized and studies of concordance in twins and families of patients affected by AS indicate that susceptibility of the disease is widely due to genetic factors (Brown et al., 1997). More recent studies reported that the major histocompatibility complex (MHC) region gives a contribution of about 20% to the heritability (Ellinghaus et al., 2016).

### HLA B27

The HLA-B27 is the allele most associated with AS, as well as with other types of SpA (Breban et al., 2015; Lin and Gong, 2017). Most AS patients express HLA-B27 (90%) and this allele is responsible for up to 28% of the etiology of SpA. Nevertheless, less than 5% of HLA-B27 positive people in the general population develop this disease (Reveille, 2011). Much research has shown a higher prevalence of HLA-B27 in males than in females. Recently, a link between HLA-27 expression and high concentrations of testosterone was demonstrated (Akassou and Bakri, 2018). HLA-B27 is highly polymorphic and several sub-alleles with different contributions to susceptibility to SpA have been identified. Of these subtypes, B^∗^2705 is found most frequently in the Caucasian population and has a strong association with AS and PsA; on the contrary, HLA-B^∗^2706, detected in Southeast Asia, and B^∗^2709, present in Sardinia, do not seem to be related to SpA (Haroon et al., 2017).

The main function of HLA-B27 is the presentation of intracellular peptides to cytotoxic (CD8-positive) T lymphocytes and multiple theories about its pathogenic mechanisms have been suggested. These hypotheses include the presentation of “arthritogenic” peptide recognized by the T-cell receptor (TCR) of autoreactive CD8^+^ T cells; cell surface HLA-B27 homodimers recognition by natural killer (NK) receptors; accumulation of misfolded HLAB27 in the endoplasmic reticulum during protein biosynthesis leading to inflammatory response; failed elimination of bacteria or virus with consequent intracellular microbial survival and prolonged abnormal immune system activation (Haroon et al., 2017). Recently, it has been described that HLA-B27 could perturbate the composition of the gut microbiota, leading to loss of mucosal tolerance and activation of aberrant inflammatory response. Significant differences in microbiota composition were detected between HLA-B27-positive and HLA-B27-negative SpA patients, with increased *R. mucilaginosa* and *E. lenta*, and low levels of *Bifidobacterium* and *Odoribacter* in HLA-B27 positive patients, similar to what has been reported in patients with ileal Crohn’s disease (CD) and ulcerative colitis (UC) (Chimenti et al., 2018; Lim et al., 2018). Moreover, a recent metabolome analysis in HLA-B27 transgenic rats has shown a perturbation in levels of short-chain fatty acids and other microbial metabolites during gut inflammation (Asquit et al., 2017). Microbial dysbiosis and perturbed microbial metabolic function are associated with inflammatory pathways (IFNg, TNF, and IL-23/IL-17) and with imbalance between Tregs, Th1, Th2, and Th17 cells, which may lead to chronic inflammation in the joint, skin, or gut as well as a loss of tolerance for non-pathogenous (Asquit et al., 2017; Gill et al., 2019). Even though HLA-B27 plays an undisputedly critical role in AS pathogenesis, only 1–3% of HLA-B27-positive people develop the disease, and not all AS patients carry the HLA-B27 antigen, advising that other genes may be involved in the development of the pathology (Chimenti et al., 2018; Gill et al., 2019).

### Other HLA Genes

Different mechanisms have been advanced to explain SpA susceptibility, such as large variants (deletions, duplications, inversions), single nucleotide polymorphisms (SNPs), gene-gene, and gene-environment interactions. In the last decades, the advent of genome-wide association studies (GWASs) has improved our understanding of SpA disease pathogenesis. HLA-B51, mostly associated with Behçet’s disease, seems to be a risk variant for AS, as well as HLA-B40, most specifically the B^∗^40:01 allele (a DNA-defined allele that corresponds to HLA-B60 at the serologically defined or protein level) was increased in B27-positive patients with AS (Clarke and Vyse, 2009; Dougados and Baeten, 2011). HLA-B27 and HLA-B60 genes are located on the same chromosome but they could be involved in different antigen-triggered pathologic pathways. Therefore, the epistatic effects between HLA-B27 and HLA-B60 may be due to the similar downstream T-cell mediated immune response (Wei et al., 2015). Moreover, HLA-Cw^∗^0702 is associated with axial PsA, HLA-DQ3 is involved in both PsA disease and its progression (Chimenti et al., 2020b). Although some HLA-B/C haplotypes are ancestral, explaining their over-expression in SpA, the role of these alleles in determining the risk and the clinical expression of disease is still under investigation (Queiro et al., 2006). Recent studies investigated the MHC class I chain A related (MICA), which mediates the activation of natural killer cells, γδT cells, and αβCD8^+^ T cells. MICA polymorphisms could be associated with the susceptibility to both PsO and PsA, AS, IBD, and Behçet‘s disease (Wang and Zhou, 2015). MICA-129 Val/Val polymorphism has been identified as protective against radiographic axial PsA, probably due to a low affinity for its receptor and a lower inflammation on sacroiliac joint (Wang and Zhou, 2015; Fechtenbaum et al., 2019). Nevertheless, a defining contribution of MICA, as well as other non-HLA MCH genes (e.g., TNF, TAP1, TAP2, and LMP2) to AS susceptibility has not yet been established and could be confounded by the linkage disequilibrium known to exist in this region (Fechtenbaum et al., 2019).

### Non-HLA Genes

Outside the MHC region, other genes have been identified as risk factor for SpA. Among them, evidence from the literature highlights the ERAP1 and 2, that show epistasis with HLA-B^∗^27. ERAP1 is associated with AS only in individuals carrying the HLA-B27 or HLA-B^∗^40:01 alleles (Wang and Zhou, 2015), while interaction between ERAP2 and AS is present in both HLA-B27 positive and negative disease, suggesting their different functional mechanism in causing AS (Reveille, 2014). The main function of ERAP1 and 2 products is to trim peptides to an optimal length for MHC class I binding and presentation (Cortes et al., 2015). ERAP1 variants cause a modification in three-dimensional structure of protein and could also influence gene expression. It was shown a higher expression of ERAP1 in dendritic cells of AS patients compared to healthy controls, suggesting that overexpression of ERAP1 could promote the disease (Robinson et al., 2015). Loss-of-function variants of ERAP1 lead to an aberrant peptides’ presentation, influencing dimerization or misfolding of HLA-B27 and contributing to disease pathogenesis, although to date the exact mechanism is unclear. The second function of ERAP1 is the cleavage of cell surface receptors for the proinflammatory cytokines IL-1 (IL-1R2), IL-6 (IL-6Rα) and TNF (TNFR1), downregulating their signaling (Chimenti et al., 2018). ERAP1 and ERAP2 haplotype (concerning *rs27044*, *rs30187*, and *rs2549782* SNPs) are associated with familial AS (Saveanu et al., 2005; Evans et al., 2011) while *rs27037*, *rs27044*, and *rs30187* SNPs are involved in syndesmophytes formation and AS severity (Saveanu et al., 2005; Tsui et al., 2010; Evans et al., 2011). In contrast, ERAP2 variant *rs2248374* seems to cause a loss of ERAP2 protein by reducing MHC class I surface expression in cell lines, being potentially protective for AS (Wang et al., 2012; Paladini et al., 2019). Another gene described as involved in AS susceptibility is RUNX3, involved in differentiation of cytotoxic-lineage T cells into phenotypically mature CD8^+^ T cells, which have been implicated in the pathogenesis of AS. Moreover, RUNX3 regulated other immunological cells and through TGF-β signaling pathway could drive the imbalance of Th17/Treg in AS (Vecellio et al., 2019). Three other AS–associated genes (EOMES, IL7R, and ZMIZ1) were also identified as impacting on variation in CD8^+^ lymphocyte counts and differentiations. Probably other mechanisms besides the effect on lymphocytes are underlying but they are still unknown (Evans et al., 2011). RUNX3 polymorphism (*rs6600247*) is associated with lower CD8^+^ T cell counts, causing an altered antigen presentation, while the opposite occurred with IL7R haplotype rs991570, suggesting an unknown mechanism related to the IL7/RUNX3 pathway (Roberts et al., 2016; Vecellio et al., 2019). Previously, it was identified that other AS-associated SNPs, *rs4648889* and *rs4265380*, located upstream of the RUNX3 gene, might have a regulatory impact respectively on CD8^+^ T cell and monocytes and similar genetic associations have been also described in PsA (Di Meglio et al., 2011). In AS, such as in IBD, genetic associations have also been reported at TBX21, encoding the transcriptional factor T-bet that control the functional differentiation of many cell types and is overexpressed in AS CD8 T-cells and NK cells. Moreover, expression of TBX21 and T-bet is higher in AS patients than controls and homozygosity for the rs11657479 risk allele increases T-bet expression (O’Rielly and Rahman, 2013; Lau et al., 2017). Associations with other genes (DEFB4, CDKAL1, KIF21B, ORMDL3, MST1, and PSMG) have been proposed but their relevance in AS has not been confirmed (Reveille et al., 2010; Aita et al., 2018).

### Cytokines

Several lines of evidence suggested a relevant role of IL-17 and IL-23 in the pathogenesis of AS. association between the IL-23R locus and AS have been demonstrated, as well as genes related to the IL-17 pathway and risk alleles for AS. The identification of protective alleles in IL23R that result in reduced phosphorylation of signal transducer and activator of transcription 3 (STAT3) and in impairment production of IL-17 supports the hypothesis that activation of the IL-23–IL-17 pathway is controlled at a genetic level (Gravellese and Schett, 2018). Association between IL23R variants and SpA, reported by GWAS studies, has strengthened the importance of IL-17-IL23 axis SpA pathogenesis. For example, the *rs11209032* SNP, located within the intergenic region between IL23R and IL12RB2, might influence Th1-cell number and correlates with disease susceptibility (Roberts et al., 2016). Also, the variant allele of *rs11209026* SNP, previously associated with IBD, modifies the interaction between IL23R and its signaling partner, JAK-2 kinase, with a protective effect. Indeed, carriers of this allele showed a decreased IL-17 and IL-22, as well as a reduction of circulating Th17 cells (Di Meglio et al., 2011). Other variants within genes encoding proteins crucial for Th-17 signaling (TRAF3IP2, TYK2, STAT3, SOCS1, IRF4, and KLF4) have been investigated but further functional studies are needed to explain how these variants contribute to susceptibility and phenotypic expression of SpA. Further SpA-associated gene pathways include IFNs, IL-1, and TNFα. IFN is a key early mediator of inflammation, involved in production of proinflammatory cytokines, e.g., TNFα and IL-1, as well as in activation of NFkB signaling (Gravellese and Schett, 2018). Dysregulated NFkB activation may lead to the transcription of several target genes contributing to SpA pathogenesis. Multiple genetic loci involved in NFkB signaling (TNFRSF1A-LTBR, TRADD, TBKBP1, CARD9, and PTGER4) have been investigated in AS. Particularly, CARD9 could promote the production of IL-17 and IL-23 and, through the indirect activation of PTGER4, influence the bone ossification and radiographic progression observed in AS (Di Meglio et al., 2011; Roberts et al., 2016). Variations within some genes encoding proteins for IFN signaling have been evidenced in GWAS analysis, mostly in patients affected by psoriasis but could have a significance also in PsA and AS (Di Meglio et al., 2011). INF is a key early mediator of inflammation, determining the production of proinflammatory cytokines, as TNF-α and interleukin (IL)-1, and influencing the activation of NFkB signaling. SNPs in TNFα gene have been identified as potentially associated with SpA. In particular, a specific *rs1799964*/*rs1800629* haplotype exerts a protective role for SpA, mainly for AS and in HLA-B27 positive subjects, as demonstrated by the reduction of TNFα release. However, the relation between SNPs of genes involved in TNFα signaling and AS has shown controversial results, maybe related to the differences in the ethnic origin or to the number of the individuals under study (Roberts et al., 2016). SNPs in IL-1 gene cluster were reported to be associated with AS, with greater focus on IL-1A and IL-1R2 genes. Nevertheless, the contribution of IL-1 in AS susceptibility is likely to be limited, as evidenced by inefficacy of IL-1 receptor antagonist in AS treatment (Sims et al., 2008; Aita et al., 2018). Based on previous published data on the association of TLRs genes with autoimmune disease, research work has been carried out on their role in AS. Toll-like receptors (TLRs) have an important role in the mechanism of innate immunity and may influence inflammatory responses (Oliveira-Toré et al., 2019). Furthermore, they are involved in the activation of adaptive immune system upregulating costimulatory molecules of the antigen-presenting cells and play a role in the self-sustained inflammatory cycle and progression of chronic diseases (Oliveira-Toré et al., 2019). Polymorphisms in TLR genes could contribute to the susceptibility to SpA. A recent study pointed out that TLR2 gene rs5743708*A polymorphism increased the chance of developing SpA. In addition, high levels of IL-12 were found in the presence of polymorphisms in TLR2 (*rs5743708*) and TLR9 (*rs5743836*) genes, while TLR9 *rs187084* was associated with increased production of IFNγ and TNFα. These polymorphisms contribute to potentiate the Th1, Th2, and Th17 immune response seen in SpA, which may confer to individuals carrying the variant alleles a predisposition to the development of SpA (Oliveira-Toré et al., 2019).

## Epigenetic Mechanisms in Spondyloarthritis

**FIGURE 1:**
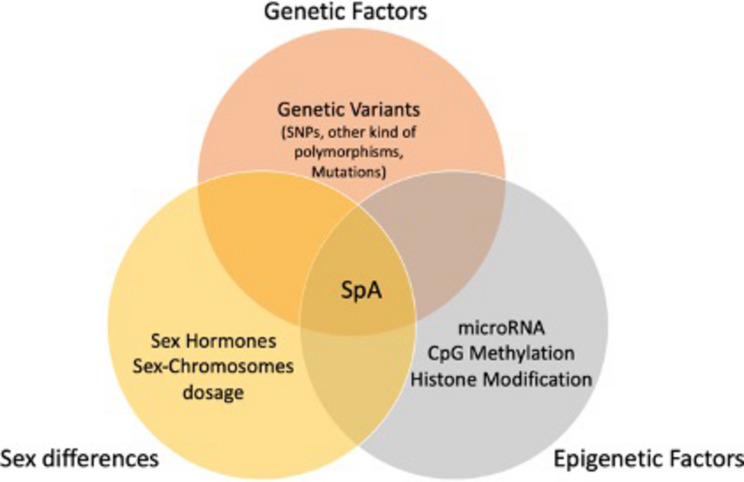
Interaction among genetic and epigenetic factors and sex in Spondyloarthritis (SpA).

Not only genetic variants but also epigenetic mechanisms, such as DNA methylation, histone modification and non-coding RNAs, have shown in the last years to be particularly relevant to explain the SpA pathogenesis. The term epigenetics refers to all mechanisms that produce a change in the gene expression, without modifying the DNA sequence. In recent decades, the interest in epigenetic mechanisms increased due to the evidence that their alterations are present in many diseases, including autoimmune diseases. In SpA, alterations of histone H3 (H3K27ac and H3K4me1) seem to be correlated with RUNX3 expression and the decrease of CD8^+^ T cell in the presence of *rs4648889* SNP variant (Cherqaoui et al., 2020). In addition, H3K4me1 methylation regulates the activity of IL23R gene. Among epigenetic mechanisms, miRNAs represent one of the most interesting examples. These small molecules of non-coding RNAs can regulate the expression of multiple genes at a post-transcriptional level by inhibiting translation or inducing messenger RNA degradation. Significant alterations in miRNA expression have been observed in axSpA patients, showing a lower expression of 14 miRNAs in comparison with healthy controls (Prajzlerová et al., 2017; Cherqaoui et al., 2020). Interestingly, most of these miRNAs are involved in osteoblast differentiation or the Wnt signaling pathway while only miR-625-3p was significantly different in nr-AxSpA patients compared to controls. Moreover, also the genetic variability of miRNAs seems to be involved with autoimmune disorders susceptibility, and several associations were already reported (Latini et al., 2017). Until now, however, there is only little evidence of associations with PsA. For example, a Chinese study reported an association between common polymorphisms in mir-146a and mir-499 genes and ankylosing spondylitis (Xu et al., 2015). As already mentioned, epigenetics could play a role in the interactions between genetic and environmental susceptibility factors and might favor SpA development. DNA methyltransferase (DNMT) 3A and 3B are involved in genomic imprinting and X-chromosome inactivation, which may in part explain the different distribution of SpA according to gender (Hanson and Brown, 2017; Hao et al., 2017). Variants in DNMT3A, DNMT3B, and DNMT3L genes have been recently associated with AS, and different methylated positions (DMPs), that can influence HLA-B27, are identified (Ellinghaus et al., 2016; Cherqaoui et al., 2020). In addition, an increased level of Histone Deacetylase 3 (HDAC3), which regulates NF-kB activity, was detected in AS patients (Jiang and Wang, 2016). Additional genetic and epigenetic mechanisms, such as genomic imprinting, could explain the gender difference in inheritance of SpA (Rahman et al., 1999). Genomic imprinting is a normal process that differentially regulates the expression of specific genes depending on the sex of the transmitting parent. Already more than twenty years ago, a higher penetrance of PsO was reported if the father was affected and a similar phenomenon was observed among PsA patients (Burden et al., 1998; Rahman et al., 1999; Karason et al., 2003). Later a significant linkage on chromosome 16q was noted only after conditioning for paternal transmission, suggesting that genetic imprinting may play a role in the inheritance pattern of psoriasis and PsA (Karason et al., 2003; Eder et al., 2012). However, despite some encouraging advances in SpA epigenetic studies, data are still lacking and further studies are needed. Table 1 summary of genes whose variability is involved in SpA. Figure 1 summarizes the interaction among genetic and epigenetic factors and sex in SpA.

**TABLE 1:** Genes whose variability is involved in Spondyloarthritis.

Genes	Chromosome	Function	Evidence of association	References
HLA-B27	6p21.3	Antigen presentation	AS, axial-PsA, enthesitis, dactylitis, uveitis	Ito et al., 2001; Reveille, 2011; Tyagi et al., 2012; Vecellio et al., 2019; Chimenti et al., 2020a
HLA-B27:05:02	6p21.3	Antigen presentation	AS, symmetric sacroiliitis, enthesitis, dactylitis, uveitis	Ito et al., 2001; Reveille, 2011; Vecellio et al., 2019; Chimenti et al., 2020a
HLA-B38	6p21.31	Antigen presentation	Peripheral PsA	Clarke and Vyse, 2009
HLA-B39	6p21.31	Antigen presentation	Peripheral PsA	Clarke and Vyse, 2009
HLA-B60 (B*4001)	6p21.3	Antigen presentation	AS, PsA	Wei et al., 2015
HLA-B15	6p21.31	Antigen presentation	AS, CD	Clarke and Vyse, 2009
HLA-C06:02	6p21.3	Antigen presentation	PsA, PsO	Chimenti et al., 2018
HLA-C07:01:01	6p21.3	Antigen presentation	Axial PsA, sacroiliitis, dactylitis	Reveille, 2011
MICA	6p21.33	NK and T cells activation	PsA, PsO	Wang and Zhou, 2015
IL-1R2	2q11-12	Interferon signaling	AS, uveitis, UC	O’Rielly and Rahman, 2013
IL-1 complex (IL-1A, IL-1B, IL1-RN)	2q14.1	Th1 response	AS, peripheral PsA	Clarke and Vyse, 2009
IL-12B	5q33.3	Th17 signaling	AS, PsA, PsO	Burden et al., 1998; Vecellio et al., 2019
IL-17A	6p12.2	Th17 signaling	AS, PsA	Burden et al., 1998; Vecellio et al., 2019
IL-17F	6p12.2	Th17 signaling	AS, PsA	Burden et al., 1998; Vecellio et al., 2019
IL-23R	1p31.3	Th17 signaling	AS, peripheral and erosive PsA, PsO, uveitis, CD, UC	Reveille et al., 2010; Gravellese and Schett, 2018
IL-23A	12q13.3	Th17 signaling	PsA, PsO, uveitis	Burden et al., 1998
IL-7R		Antigen presentation	AS, UC	Tsui et al., 2010
TNF-A	6q21.3	NFkB signaling	AS, PsA	Tsui et al., 2010
TBKBP1	17q21.32	NFkB activation and signaling	AS	Vecellio et al., 2019
TNFRSF1A-LTBR	12p13	NFkB activation and signaling	AS	Vecellio et al., 2019
ERAP1	5q15	Antigen presentation	AS, PsA, PsO, uveitis, CD, UC	Reveille et al., 2010
TBX21	17q21.32	Th1 cell expression	AS, CD	Reveille et al., 2010
FBXL19	16p11.2	NFkB activation and signaling	PsA, PsO	Vecellio et al., 2019
KIF21B	1q31	Unknown	AS, uveitis, CD, UC	Vecellio et al., 2019
CARD9	9q34.3	NFkB activation and signaling	AS, CD, UC	Gravellese and Schett, 2018
PTGER4	5p13.1	Th17 signaling	AS, CD	Vecellio et al., 2019
IRAK1	Xq28	NFkB signaling	PsA	Chimenti et al., 2019b
ANTXR2	4q21	Bone remodeling	AS	Vecellio et al., 2019
STAT3	17q21	Th17 signaling	AS, CD, UC	Berlinberg and Kuhn, 2020
CYP2D6	22q13.1	unknown	AS, CD, UC	Berlinberg and Kuhn, 2020
ANKH	5p15.2	Bone remodeling	AS	Queiro et al., 2013
TLR4	9q32-33	NFkB signaling	AS, PsA, uSpA	O’Rielly and Rahman, 2013
TLR2	4q31.3	NFkB signaling	AS, PsA, uSpA	O’Rielly and Rahman, 2013
TLR9	3p21.2	NFkB signaling	AS, PsA	O’Rielly and Rahman, 2013
TYK2	19P13.2	IFN and NFkB activation and signaling	PsA, PsO	Vecellio et al., 2019
KIR3DL1	19q13.4	NK and T cells activation	AS, PsO, PsA	Chimenti et al., 2019b
ADAM33	20p13	Cell-cell and cell-matrix interactions	PsA, PsO	Chimenti et al., 2018
TNIP1	5q32- q33.1	NFkB activation and signaling	PsA, PsO	Vecellio et al., 2019
RUNX3	1p36	Antigen presentation	AS, PsO	Vecellio et al., 2019
EOMES	3p24.1	Unknown	AS	Tsui et al., 2010
ZMIZ1	10q22.3	Unknown	AS, PsO, CD, UC	Tsui et al., 2010
ANO6	12q12	Bone remodeling	AS	Vecellio et al., 2019
DMP1	4q22.1	Bone remodeling	AS	Mori et al., 2000
HAPLN1	5q14	Bone remodeling	AS	Vecellio et al., 2019

*HLA, human leukocyte antigen; NK, Natural Killer cells; AS, Ankylosing Spondylitis; PsA, psoriatic arthritis; PsO, psoriasis; UC, ulcerative colitis; CD, Crohn disease.*

## Gender Differences in Clinical Phenotypes in Axial SpA

Several sex related differences have been described among the various subtypes of SpA. These can be divided based on joint manifestations. Differences related to gender on joint manifestations are summarized in Table 2. Regarding axial-SpA, relevant differences can be highlighted among gender (Rudwaleit et al., 2009; Conigliaro et al., 2016). First of all, a male predominance (3:1) has been found in the radiographic form while an equal sex distribution is registered in the nr-axSpA (1,03:1) (van der Linden et al., 1984; Rudwaleit et al., 2009). In this condition, disease burden appears to be similar for both sexes (Kennedy et al., 1993; Ward et al., 2009; Baumberger, 2017; Rusman et al., 2020). However, differences emerged concerning clinical and disease activity distribution. Indeed, men seem to display a higher radiological damage and a higher radiological progression (as highlighted with Bath Ankylosing Spondylitis Radiology Index and modified Stoke Ankylosing Spondylitis Spine), showing more marked radiographic spinal changes and worse hip involvement than women (Kennedy et al., 1993; Rusman et al., 2018). Confirming this, Lee et al. (2007) demonstrated a more severe radiographic damage among men who showed higher median BASRI−spine scores both in unadjusted and adjusted analyses for age and disease duration. On the other hand, the signs of peripheral involvements such as dactylitis, enthesitis, or swollen joint count appear to be significantly more prevalent in women (Kennedy et al., 1993; Rusman et al., 2020). Extra-articular manifestations also appear with a higher rate in female patients (Landi et al., 2016). Concerning acute anterior uveitis, a recent review reported a slightly higher predominance in women (M/F = 28/33%) (Zeboulon et al., 2008), as in patients affected by IBD (Stolwijk et al., 2015; Landi et al., 2016) or PsO (M/F = 25/33%) (Stolwijk et al., 2015; Landi et al., 2016) studies report a higher predominance in female patients. Regarding comorbidities such as cardiovascular diseases (CVD) and osteoporosis (Op), non-conclusive data are available on gender differences; evidences on CVD are scarce while a study on young males reported that 51% had a low bone mineral density (BMD) and 15% had Op (Zarco et al., 2015), another study concluded that male patients have a 4 times greater risk for low BMD compared with females (van der Weijden et al., 2012). These latter data are in contrast with those regarding the general population, where Op is typically prevalent in post-menopausal women (van der Weijden et al., 2012). Furthermore, CRP levels appear to be significantly higher, at baseline levels, in male patients compared with females, while no conclusive sex differences have been found for ESR (Jordan and Cooper, 2002; van der Weijden et al., 2011). Finally, women show higher disease activity, mostly in terms of subjective measures, reporting worse baseline BASDAI scores (in particular total and nocturnal back pain, duration of morning stiffness, and fatigue) as widely described in many studies (van der Horst-Bruinsma et al., 2013; Rubio Vargas et al., 2016; Kilic et al., 2017) while just a few studies described differences for baseline scores, being concordantly lower in females, of BASFI (van der Horst-Bruinsma et al., 2013; Rubio Vargas et al., 2016) and ASDAS-CRP (Webers et al., 2016). At the same time, female patients display less improvement in BASDAI, BASFI, and ASDAS-CRP scores after treatment (van der Horst-Bruinsma et al., 2013; Neuenschwander et al., 2020). In addition, women report worse quality of life when the Ankylosing Spondylitis Quality of Life questionnaire (ASQoL) and the Assessment of SpondyloArthritis international Society Health Index (ASA-HI) were used while no gender differences were found using the EuroQoL and the SF-36 Health Survey (Kennedy et al., 1993; Roussou and Sultana, 2011; Tournadre et al., 2013; Lubrano et al., 2017; Ibáñez Vodnizza et al., 2020; Neuenschwander et al., 2020). In summary, men with ax-SpA present higher objective markers of inflammation associated with radiological progression, although severe ankylosis also occurs in females. Nonetheless, women generally have higher subjective indicators of disease activity and, in addition, more peripheral involvement and extra-articular manifestations. As for comorbidities, osteoporosis has an unexpectedly high prevalence in young male patients. Concerning sex related differences in Entheropatic arthritis (EA), few data are available in literature and a small number of studies explored this topic, reporting axial manifestations as more frequently present in male patients than in females and women appear to be more likely to develop both type 1 and type 2 arthropathy (Dougados et al., 1991; Orchard et al., 1998; Turkcapar et al., 2006; Rodríguez-Reyna et al., 2009; Yüksel et al., 2011; Peluso et al., 2013; Picchianti-Diamanti et al., 2020). On the contrary, several findings suggest the presence of sex-related differences in PsA patients. It is reported that male patients have a high prevalence of axial disease and a high frequency of HLA-B27 antigens positivity, while women tend to have a more erosive disease, higher number of swollen joint count and a higher prevalence of disability (Gladman et al., 1992; Queiro et al., 2001; Wallenius et al., 2009; Queiro et al., 2013). Eder et al. (2013) demonstrated, on a large cohort of PsA patients, that axial involvement is more frequent in men as well as radiographic damage in both axial and peripheral joints (assessed with the modified Stokes Ankylosing Spondylitis Spine Score and the modified Steinbrocker score), while women reported with a higher frequency functional limitation and impaired quality of life (reporting higher scores in HAQ and BASFI and lower in the SF-36-PCS). In addition, differences were found in the pattern of arthritis at onset: women presented more frequently with the polyarticular subtype while men presented with olygoarthritis. On the other hand, the authors reported no significant differences in active and swollen joint counts, the presence of dactylitis, inflammatory spinal pain, and PASI score even if psoriatic nail lesions were more frequently found in men (Queiro et al., 2001).

**TABLE 2:** Clinical differences related to gender in Ankylosing Spondylitis.

Sex related differences in AS	
Radiological progression Radiological damage	**↑**In men
Axial involvement	**↑**In men
Peripheral involvement	**↑** In women
Swollen joint count	**↑**In women
Osteoporosis	**↑** In men
Disease activity scores	
BASDAI, baseline	**↑** In women
BASDAI, improvement	
BASFI, improvement	**↑**In men
AS DAS, improvement	
ASQoL AS AS-HI	**↑**In men
EuroQoL	No differences

*AS, Ankylosing Spondylitis; BASDAI, Bath Ankylosing Spondylitis Disease Activity Index; BASFI, Bath Ankylosing Spondylitis Functional Index; ASDAS, Ankylosing Spondylitis Disease Activity Score; ASQoL, Assessment of Spondyloarthritis Quality of life; ASA-HI, Assessment of Spondyloarthritis international Society Health Index; EQ-5D, EuroQoL-5D; SF-36 HS, SF-36 Health Status.*

## Gender and Genetic Polymorphisms

Accumulating evidence suggests that gender differences in severity of SpA and treatment response could be due to genetic, immunological and hormonal factors (Brown et al., 1997). Female karyotype includes two X chromosomes, one of which is randomly silenced during embryogenesis. However, about 15% of the genes escape inactivation, leading to overexpression of some X-linked genes in females. The X chromosome encodes several immune-related genes, such as TLR7, TLR8, FOXP3, and IL-2 receptor gamma, whose overexpression may influence the immune response in a sex-dependent manner. For instance, male carriers of TLR9 *rs187084* variant allele have an increased production of IFN-γ and TNFα, and women with rs5743836*C allele have a higher risk of developing SpA (Oliveira-Toré et al., 2019). Moreover, in contrast to the Y chromosome, the X chromosome is highly enriched in miRNAs, and some of them are reported to play a role in immunity or autoimmunity (Ortona et al., 2016). Different variants of the ANKH gene, which encode for a protein involved in osteogenesis and structural damage in axSpA, are expressed in men and women with AS. In women, AS seemed to be associated with genetic markers at the 5′ end of the ANKH gene, whereas in men with genetic markers at the 3′ end of this gene (Tsui et al., 2005). In this latter region, haplotype analysis has shown several SNPs associated with AS, supporting the greater predisposition of the male sex to develop AS. Moreover, different haplotypes combinations were significantly associated with AS in men (including *rs26307* and *rs27356* SNPs) and in women (including *rs28006* and *rs25957)* SNPs) (Ortona et al., 2016). Furthermore, one specific haplotype in TNAP (tissue-non-specific alkaline phosphatase) gene, which interplays with the ANKH gene in ossification, was associated with AS in men but not in women (Tsui et al., 2005). A recent study on AS patients identified an upregulation of IL-17RA gene expression in men compared with women, as well as greater levels of TNFα, IL-18, IL-17A, and peripheral T-helper 17 cells. On the contrary, IL-6 levels were higher in women than in men (Tsui et al., 2007; Gracey et al., 2016). Sex differences in the expression of these genes could explain the higher prevalence and radiographic progression of AS in men compared with women and might be also influenced by sex hormones (Huang et al., 2012; Cutolo and Straub, 2020). Although few studies have investigated sex differences separately, several analyses have assessed sex as a possible predictor in relation to treatment efficacy and drug survival. Recent studies have shown that women tended to discontinue TNFi earlier than men both in axSpA and in PsA (Lubrano et al., 2017; Rusman et al., 2018). This might be partly explained by the lower prevalence of HLA-B27, a longer disease duration, and a greater fat mass in women than men. These factors are associated with a lower TNFi treatment response (Roussou and Sultana, 2011). Moreover, data from registries, such as the DANBIO and BSRBR, suggested that women also suffered from more side effects that have led to the discontinuation of the drugs (Saad et al., 2009; Glintborg et al., 2011). Polymorphisms within the TNF promoter region have been identified to influence clinical efficacy of etanercept in RA, AS, and PsA patients (Pierik et al., 2004; Morales-Lara et al., 2012). TNFR1A variant *rs767455/G36A* in PsA patients has been associated with a better EULAR response at 3 months to infliximab and similar results were obtained in CD patients (Pierik et al., 2004; Morales-Lara et al., 2012). On the contrary, the TNFAIP3 *rs2230926* and *rs610604 variant alleles* seem to be correlated with better TNFi response in PsO patients, while *rs6920220* and *rs610604* are associated with an improvement in the quality of life in PsA patients receiving TNFi (Tejasvi et al., 2012; Ovejero-Benito et al., 2019). One study suggested that TNFi failure can occur in AS patient carriers of variants within macrophage migration inhibitory factor (MIF) gene (*rs755622*), IL-18R gene (rs917997), IL10 (rs1800896), and TNFRSFB1 (rs4355801) (Schiotis et al., 2014). Another study has reported that female gender, elevated basal BASFI index, and being a CHUK gene *rs11591741-GC* genotype carrier are predictors of long-term non-response to TNFi in SpA patients (Polo et al., 2019). Moreover, a lower level of clinical response measured by ASDAS and BASDAI index was observed in females (Rusman et al., 2018). Different miRNAs, such as miR-146a, miR-155, miR-625-3p, and miR-29a were linked to BASDAI, reflecting disease activity in AS with spinal involvement. In addition, miR-146a-5p, miR-125a-5p, and miR-22-3p expression were correlated with the plasma cytokine levels (TNFα, IL-1β, and IL-5), CRP and ESR (Perez-Sanchez et al., 2018). Downregulation of miR-199a-5p has been associated with increased TNFα, IL-17, and IL-23 in AS, suggesting that miRNAs could distinguish SpA phenotypes responsive to inhibitors of TNFα, IL-17, or IL-23 (Berlinberg and Kuhn, 2020). Proinflammatory cytokines, such as IL-17, are decisively involved in all clinical manifestations of SpA, and different concentrations of the cytokines are observed between men and women (Ortona et al., 2016). The use of secukinumab is widely diffused in our clinical practice for SpA management (Chimenti et al., 2019b). Recently the efficacy of secukinumab has been evaluated in patients with SpA, both in PsA and in AS patients. At the multivariate analysis, there was no gender influence concerning treatment efficacy. However, gender difference was observed according to drug-survival, particularly in the AS population: males had a higher persistence rate than females; this was not demonstrated in PsA patients (Chimenti et al., 2020a).

## Gender and Hormones in Axial SpA

Since last century, the role of sex hormones has been hypothesized for the development of immune mediated diseases. Generally, estrogens have roles in both enhancing and inhibiting immune system activity, whereas androgens and progesterone exert suppressive effects (Cutolo and Straub, 2020). It has been reported that sex steroids can influence epigenetic modulation and expression of microRNAs that interfere with immune responses in autoimmune rheumatic diseases (Gracey et al., 2016). However, their role in SpA has been only partially evaluated. Estrogen has mainly anti-inflammatory effects, by inhibiting TNFα production, but contradicting results were presented (Rusman et al., 2018). This concept was previously supported by an old study demonstrating an improvement of arthritis in AS patients treated with oral estrogen and a worsening of disease in patients with low levels of estrogens (Gracey et al., 2016). Conversely, a more recent report showed neither difference in onset nor severity of AS female patients despite differences in serum estrogen concentration (Huang et al., 2012). For instance, estrogen modulates immune-related processes such as T cell differentiation and cytokine production and animal model studies have suggested that estrogen can inhibit Th17 differentiation from naïve T cells and in human 17β-estradiol (E2) levels are lower in patients with active AS than in those with inactive AS (Tyagi et al., 2012). Experimental models, such as E2-treated mice, showed a relationship among cytokines production and sex hormones: a decreased expression of TNF in the joint tissue was demonstrated supporting the inhibition of TNF production by estrogens (Ito et al., 2001). Estrogen-deficient female rats are also reported to have higher serum TNF than estrogen-supplemented animals and high and medium doses of estrogen increased the production of IL-4, IL-10, and TGF-β, whereas estrogen treatment reduced the production of inflammatory cytokines such as IFN-γ, IL-17, and IL-6. The relevant role of IL-17 in SpA pathogenesis and in particular in axial manifestation is well noted in clinical practice. A link between sex hormones and Th17 activation was demonstrated: estrogen inhibits Th17 cell. In ovariectomized DBA/1 mice with collagen-induced arthritis (CIA), E2 treatment reduces the severity of arthritis and results in fewer Th17 cells in the joints compared with controls (Andersson et al., 2015). Jeong et al. (2017) recently aimed to evaluate the effect of estrogen on the disease activity of SpA in a mouse model of SpA. The E2-treated group had significantly suppressed arthritis compared with both the ovariectomized and the sham groups. Furthermore, the expression of Dkk1 was significantly increased in E2-treated mice compared with the ovariectomized and sham groups and the expression of Wnt inhibitors was inhibited by estrogen (Jeong et al., 2017). Considering that blockade of Wnt inhibitors induced the fusion of sacroiliac joints and increased bone formation, estrogens might act in controlling Wnt pathway and used as a therapeutic target (Uderhardt et al., 2010). Unfortunately, in SpA, neither androgens and androgen receptors nor estrogen receptors polymorphisms were evaluated. Shorter CAG repeats of the androgen receptor gene presenting high levels of transactivation activity were demonstrated in a Japanese cohort of AS patients, supporting a role in male AS development (Mori et al., 2000). The role of microbiota has been previously described in SpA and its link with HLA B27 has been reported (Chimenti et al., 2018). Interestingly, women and men tend to have different gut microbiota, suggesting that sex hormones might have an effect on microflora, even if the mechanism still remains unclear (Cutolo and Straub, 2020). Sex-dependent differences in gut microbiota may lead to genetic or epigenetic changes in local gastrointestinal inflammation, systemic immunity, and susceptibility to a range of rheumatic diseases (Rizzetto et al., 2018; Cutolo and Straub, 2020). However, no progress has been made in decoding the unequivocal role of sex steroids in gut microbiota-related effects on SpA patients. Moreover, several studies in humans have highlighted that sex- related differences in the microbiota can occur across the lifespan of an individual, making results questionable (Ding and Schloss, 2014; Jaggar et al., 2019). Other relationships between gender and hormones are associated with levels of circulating adipokines. It is well known that obesity, body composition, and adipokines have an influence on differences in disease activity, progression, and response to treatment, between men and women with SpA (Rusman et al., 2018). Body fat content is higher in women than men and obesity is related to worse disease activity scores in SpA. In details, leptin, which is usually found at higher levels in overweight women and higher in women than men, was associated to spinal radiographic progression. Moreover, women also have higher circulating adiponectin levels, which is an insulin-sensitizing hormone. Positive correlations with inflammatory biomarkers, such as CRP and TNF, have been observed for leptin (Graßmann et al., 2017). Being a woman and being obese, mainly because of the body fat content, are related to a worse response to TNF-α blockers (Ibáñez Vodnizza and van der Horst-Bruinsma, 2020).

## Conclusion

Based on genetic predisposition related to HLA-B27, SpA was generally considered as a male disease. As a matter of fact, in 1949, the male:female ratio was estimated to be 10:1 for AS patients; yet recent data have established a new 2–3:1 ratio (Kennedy et al., 1993). This epidemiological difference from past to present is due to several considerations: (1) the improvement and the development of new classification criteria aiming at the inclusion of early disease phases as well as the definition of non-radiographic SpA, (2) the better knowledge of clinical manifestations of SpA in the rheumatological field but also in other disciplines, (3) the improvement of radiological techniques for diagnosis and management. However, the highest prevalence of some clinical manifestations in females with respect to male suggested differences in the pathogenesis of SpA. The relevance of genetic and epigenetic phenomena in SpA pathogenesis was highlightened in this review, as well as the role of sex hormones, supporting the need of a redefinition of clinical and therapeutic targets. bDMARDs dramatically improved the management and quality of life of patients affected by SpA. With the availability of TNFi, research interests were oriented in order to identify potential clinical variables and biomarkers able to define the best responder to these drugs (Rubio Vargas et al., 2016). Different drugs with different mechanisms of action such as Il-17 inhibitors or Jak-inhibitors may have the same effect and efficacy in both men and women.

## Author Contributions

All authors listed have made a substantial, direct and intellectual contribution to the work, and approved it for publication.

## Conflict of Interest

The authors declare that the research was conducted in the absence of any commercial or financial relationships that could be construed as a potential conflict of interest. The handling editor declared a past co-authorship with the authors RP, PT, PC, and MC.

## Publisher’s Note

All claims expressed in this article are solely those of the authors and do not necessarily represent those of their affiliated organizations, or those of the publisher, the editors and the reviewers. Any product that may be evaluated in this article, or claim that may be made by its manufacturer, is not guaranteed or endorsed by the publisher.
